# 4,4′,6,6′-Tetra-*tert*-butyl-2,2′-[1,3-diazinane-1,3-diylbis(methyl­ene)]diphenol 0.25-hydrate

**DOI:** 10.1107/S1600536812026505

**Published:** 2012-06-16

**Authors:** Manman Zhang, Li Li, Fugen Yuan, Hui Qian

**Affiliations:** a School of Chemistry and Biochemistry, University of Science and Technology of Suzhou 215009, People’s Republic of China

## Abstract

The title compound, C_34_H_54_N_2_O_2_·0.25H_2_O, the organic mol­ecule, a potential tetra­dentate ligand with a bulky phenolic donor, has overall mirror symmetry. A partially occupied water mol­ecule of solvation is present in the lattice. The six-membered 1,3-diazinane ring displays a chair conformation. An intra­molecular O—H⋯N hydrogen bond ocurs. In the crystal, mol­ecules are linked by O—H⋯O inter­actions.

## Related literature

For amino­bis­phenolato ligands in coordination chemistry, see: Wichmann *et al.* (2012 ▶). For applications of their metal complexes, see: Barroso *et al.* (2010 ▶); Wong *et al.* (2010 ▶); Kannan *et al.* (2008 ▶); Pang *et al.* (2008 ▶); Tshuva *et al.* (2001 ▶). For background to the synthetic procedure and related structures, see: Hancock *et al.* (2011 ▶); Manna *et al.* (2008 ▶); Mohanty *et al.* (2008 ▶); Guo *et al.* (2003 ▶).
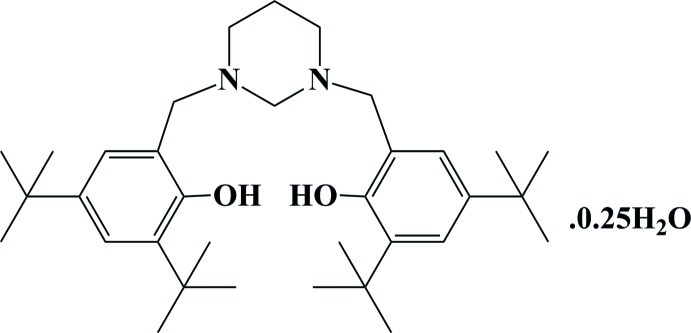


## Experimental

### 

#### Crystal data

C_34_H_54_N_2_O_2_·0.25H_2_O
*M* *_r_* = 527.30
Orthorhombic, 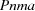

*a* = 8.7292 (8) Å
*b* = 37.428 (3) Å
*c* = 10.1806 (9) Å
*V* = 3326.1 (5) Å^3^
*Z* = 4
Mo *K*α radiation
μ = 0.07 mm^−1^
*T* = 296 K
0.33 × 0.24 × 0.16 mm

#### Data collection

Bruker SMART APEXII CCD diffractometer
29196 measured reflections
3908 independent reflections
3411 reflections with *I* > 2σ(*I*)
*R*_int_ = 0.031

#### Refinement

*R*[*F* ^2^ > 2σ(*F* ^2^)] = 0.046
*wR*(*F* ^2^) = 0.130
*S* = 1.05
3908 reflections
180 parameters
1 restraint
H-atom parameters constrained
Δρ_max_ = 0.38 e Å^−3^
Δρ_min_ = −0.31 e Å^−3^

### 

Data collection: *APEX2* (Bruker, 2009 ▶); cell refinement: *SAINT* (Bruker, 2009 ▶); data reduction: *SAINT*; program(s) used to solve structure: *SHELXS97* (Sheldrick, 2008 ▶); program(s) used to refine structure: *SHELXL97* (Sheldrick, 2008 ▶); molecular graphics: *SHELXTL* (Sheldrick, 2008 ▶); software used to prepare material for publication: *publCIF* (Westrip, 2010 ▶) and *PLATON* (Spek, 2009 ▶).

## Supplementary Material

Crystal structure: contains datablock(s) I, global. DOI: 10.1107/S1600536812026505/fj2558sup1.cif

Structure factors: contains datablock(s) I. DOI: 10.1107/S1600536812026505/fj2558Isup2.hkl

Additional supplementary materials:  crystallographic information; 3D view; checkCIF report

## Figures and Tables

**Table 1:** Hydrogen-bond geometry (Å, °)

*D*—H⋯*A*	*D*—H	H⋯*A*	*D*⋯*A*	*D*—H⋯*A*
O1—H1⋯N1	0.82	2.00	2.6880 (13)	142
O2—H2⋯O1^i^	0.85	2.22	3.036 (5)	161

Symmetry code: (i) 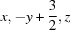.

**Fig. 1.**
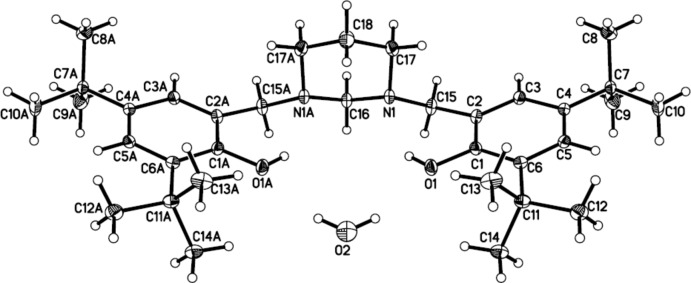
The molecular structure of the title compound with ellipsoids scaled to 30% probability.

**Fig. 2.**
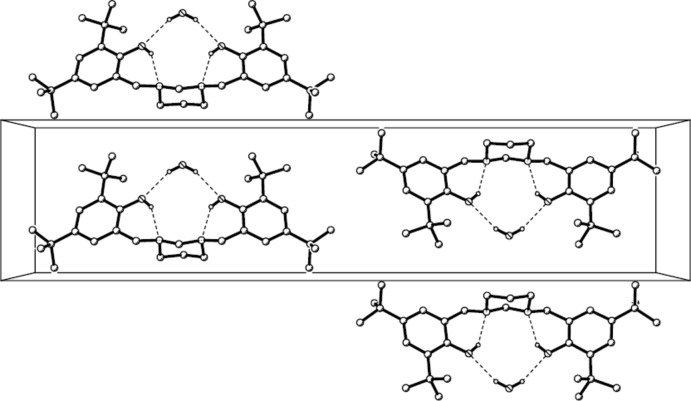
intra and inter molecular contacts (dashed line) as well as molecular packing of the title compound along c axis.

## supplementary crystallographic information

### Comment

In coordination chemistry, various ligands are used to control the environment of the metal. Especially, the electronic and steric properties of ligands are used to control the reactivity of metal species. Aminobisphenolato ligand is attractive, because its substituents at the phenolate rings as well as the position and nature of the side chain donor are easily tuneable features; thus different electronic and steric properties are available (Wichmann *et al.* 2012). Such metal complexes have been found to display catalytic activity for polymerization of cyclic esters (Hancock *et al.*, 2011) (biodegradable polymers) and polymerization of olefins (Tshuva *et al.*, 2001). They can also catalyse Tischenko reactions (Pang *et al.*, 2008), sulfoxidations (Barroso *et al.*, 2010), olefin epoxidation (Wong *et al.*, 2010), hydrogenation of ketones (Kannan *et al.*, 2008) and Mizorokie-Heck coupling reaction (Mohanty *et al.*, 2008). Generally, aminobisphenolato ligands are prepared by Mannich condensation from formaldehyde, phenol and a primary amine (Manna *et al.*, 2008; Guo *et al.*, 2003). Herein we present a new ligand, in which two substituted phenols are bridge-linked by a tetrahydropyrimidine ring. The molecules form a mirror symmetric structure, as illustrated in Scheme 1.

In the title compound, the C—N bond distances are between 1.4580 (14) to 1.4763 (15) Å whereas the bond length of C—O is 1.3763 (15) Å. The bond angles around the nitrogen atoms range from 110.35 (12)*^o^* to 112.08 (10)*^o^*, which is in agreement with those in similar structure (Guo *et al.*, 2003). Two phenolate groups are linked by a tetrahydropyrimidine ring. The overall geometry is mirror-symmetric (Fig.1). The six-member 1,3-diazacyclohexane ring displays in a chair-configuration. Compound molecules were stabilized by hydrogen bonds including intra-molecular O—H···N interaction and inter-molecular O—H···O interaction (Fig. 2).

### Experimental

The title compound was prepared as follows. To a solution of 2,4-di-butylphenol (24.77 g, 0.12 mol) in 20 ml of methanol was added 7 ml of formaldehyde(0.08 mol) and 2.0 ml 1,3-propanediamine. The mixture was refluxed for 3 d at 65 *^o^*C. In the process white precipitates were produced gradually. After being filtered and washed with methanol for 3 times, white product of C_136_H_218_N_8_O_9_ was obtained in a yield of 90.7% (based on diamine). Single crystals were grown from ethyl acetate, m.p. = 185 °C, Anal. calcd for C_136_H_218_N_8_O_9_: C, 77.44; H, 10.42; N, 5.31; Found: C, 77.02; H, 10.55; N, 5.27. IR(KBr, cm^-1^) 3434(w), 2956(*s*), 2906(*s*), 2870(*s*), 2806(*s*), 2726(*m*), 2680(*m*), 1607(*m*), 1480(*s*), 1459(*s*), 1442(*s*), 1392(*m*), 1362(*s*), 1307(*s*), 1286(w), 1235(*s*), 1204(*m*), 1189(*m*), 1167(*m*), 1123(w), 1110(*m*), 1095(*m*), 989(*m*), 883(*m*), 822(w), 797(w), 761(w), 724(w), 682(w), 460(w).

### Refinement

Tertiary Carbon H atoms were constrained to ideal geometry, with C—H = 0.98 Å and *U*_iso_(H) = 1.5*U*_eq_(C), All other H atoms were placed in geometrically idealized positions and constrained to ride on their parent atoms, with C—H = 0.93 (aromatic and alkenyl) *U_iso_*(H) = 1.2*U*_eq_(C). The dispalcement parameters for the water O atom were very large at full occupancy. When refined, its fractional occupancy converged to close to 0.25 and was then set at this value.

### Crystal data

**Table:**

C_34_H_54_N_2_O_2_·0.25H_2_O	*F*(000) = 1162
*M**_r_* = 527.30	*D*_x_ = 1.053 Mg m^−^^3^
Orthorhombic, *P**n**m**a*	Mo *K*α radiation, λ = 0.71073 Å
Hall symbol: -P 2ac 2n	Cell parameters from 9920 reflections
*a* = 8.7292 (8) Å	θ = 2.6–27.6°
*b* = 37.428 (3) Å	µ = 0.07 mm^−^^1^
*c* = 10.1806 (9) Å	*T* = 296 K
*V* = 3326.1 (5) Å^3^	Prism, colorless
*Z* = 4	0.33 × 0.24 × 0.16 mm

### Data collection

**Table:**

Bruker SMART APEXII CCD diffractometer	3411 reflections with *I* > 2σ(*I*)
Radiation source: fine-focus sealed tube	*R*_int_ = 0.031
Graphite monochromator	θ_max_ = 27.6°, θ_min_ = 1.6°
phi and ω scans	*h* = −11→11
29196 measured reflections	*k* = −48→48
3908 independent reflections	*l* = −13→13

### Refinement

**Table:**

Refinement on *F*^2^	Secondary atom site location: difference Fourier map
Least-squares matrix: full	Hydrogen site location: inferred from neighbouring sites
*R*[*F*^2^ > 2σ(*F*^2^)] = 0.046	H-atom parameters constrained
*wR*(*F*^2^) = 0.130	*w* = 1/[σ^2^(*F*_o_^2^) + (0.0634*P*)^2^ + 1.2767*P*] where *P* = (*F*_o_^2^ + 2*F*_c_^2^)/3
*S* = 1.05	(Δ/σ)_max_ = 0.038
3908 reflections	Δρ_max_ = 0.38 e Å^−^^3^
180 parameters	Δρ_min_ = −0.31 e Å^−^^3^
1 restraint	Extinction correction: *SHELXL97* (Sheldrick, 2008), Fc^*^=kFc[1+0.001xFc^2^λ^3^/sin(2θ)]^-1/4^
Primary atom site location: structure-invariant direct methods	Extinction coefficient: 0.0034 (7)

### Special details

**Table:**

Geometry. All e.s.d.'s (except the e.s.d. in the dihedral angle between two l.s. planes) are estimated using the full covariance matrix. The cell e.s.d.'s are taken into account individually in the estimation of e.s.d.'s in distances, angles and torsion angles; correlations between e.s.d.'s in cell parameters are only used when they are defined by crystal symmetry. An approximate (isotropic) treatment of cell e.s.d.'s is used for estimating e.s.d.'s involving l.s. planes.
Refinement. Refinement of *F*^2^ against ALL reflections. The weighted *R*-factor *wR* and goodness of fit *S* are based on *F*^2^, conventional *R*-factors *R* are based on *F*, with *F* set to zero for negative *F*^2^. The threshold expression of *F*^2^ > σ(*F*^2^) is used only for calculating *R*-factors(gt) *etc*. and is not relevant to the choice of reflections for refinement. *R*-factors based on *F*^2^ are statistically about twice as large as those based on *F*, and *R*- factors based on ALL data will be even larger.

### Fractional atomic coordinates and isotropic or equivalent isotropic displacement parameters (Å^2^)

**Table:**

	*x*	*y*	*z*	*U*_iso_*/*U*_eq_	Occ. (<1)
N1	0.24037 (12)	0.78185 (2)	0.40084 (10)	0.0226 (2)	
O1	0.50878 (10)	0.81077 (2)	0.47464 (9)	0.0290 (2)	
H1	0.4572	0.7956	0.4369	0.044*	
O2	0.7322 (8)	0.7500	0.5233 (7)	0.0500 (15)*	0.25
H2	0.6880	0.7299	0.5133	0.075*	0.25
C1	0.44073 (13)	0.84373 (3)	0.46035 (11)	0.0218 (2)	
C2	0.30352 (13)	0.84657 (3)	0.38874 (11)	0.0224 (2)	
C3	0.23273 (13)	0.87966 (3)	0.37546 (12)	0.0228 (2)	
H3A	0.1408	0.8812	0.3297	0.027*	
C4	0.29645 (13)	0.91060 (3)	0.42917 (11)	0.0215 (2)	
C5	0.43413 (13)	0.90681 (3)	0.49783 (11)	0.0220 (2)	
H5A	0.4783	0.9272	0.5335	0.026*	
C6	0.50974 (13)	0.87414 (3)	0.51622 (11)	0.0211 (2)	
C7	0.21487 (13)	0.94668 (3)	0.41266 (12)	0.0248 (3)	
C8	0.05775 (16)	0.94463 (4)	0.47962 (16)	0.0397 (3)	
H8A	0.0056	0.9671	0.4697	0.060*	
H8B	0.0712	0.9395	0.5713	0.060*	
H8C	−0.0019	0.9260	0.4397	0.060*	
C9	0.19124 (19)	0.95464 (4)	0.26617 (16)	0.0429 (4)	
H9A	0.2890	0.9560	0.2232	0.064*	
H9B	0.1386	0.9770	0.2565	0.064*	
H9C	0.1314	0.9359	0.2272	0.064*	
C10	0.30511 (17)	0.97759 (4)	0.4729 (2)	0.0484 (4)	
H10A	0.4038	0.9792	0.4317	0.073*	
H10B	0.3180	0.9735	0.5654	0.073*	
H10C	0.2503	0.9995	0.4596	0.073*	
C11	0.66220 (13)	0.87198 (3)	0.59218 (11)	0.0240 (3)	
C12	0.71429 (15)	0.90887 (4)	0.64144 (14)	0.0334 (3)	
H12A	0.6375	0.9187	0.6985	0.050*	
H12B	0.7290	0.9245	0.5678	0.050*	
H12C	0.8089	0.9065	0.6887	0.050*	
C13	0.64494 (16)	0.84776 (4)	0.71373 (13)	0.0365 (3)	
H13A	0.5661	0.8571	0.7698	0.055*	
H13B	0.7401	0.8470	0.7609	0.055*	
H13C	0.6178	0.8241	0.6862	0.055*	
C14	0.78871 (14)	0.85726 (4)	0.50186 (12)	0.0299 (3)	
H14A	0.7992	0.8725	0.4265	0.045*	
H14B	0.7619	0.8336	0.4738	0.045*	
H14C	0.8840	0.8565	0.5489	0.045*	
C15	0.23842 (15)	0.81429 (3)	0.31813 (12)	0.0261 (3)	
H15A	0.1338	0.8193	0.2919	0.031*	
H15B	0.2976	0.8099	0.2391	0.031*	
C16	0.2173 (2)	0.7500	0.32086 (16)	0.0228 (3)	
H16A	0.2891	0.7500	0.2481	0.027*	
H16B	0.1143	0.7500	0.2851	0.027*	
C17	0.12559 (16)	0.78340 (3)	0.50660 (13)	0.0310 (3)	
H17A	0.0237	0.7850	0.4690	0.037*	
H17B	0.1426	0.8045	0.5601	0.037*	
C18	0.1380 (3)	0.7500	0.59142 (19)	0.0369 (4)	
H18A	0.2354	0.7500	0.6373	0.044*	
H18B	0.0569	0.7500	0.6565	0.044*	

### Atomic displacement parameters (Å^2^)

**Table:**

	*U*^11^	*U*^22^	*U*^33^	*U*^12^	*U*^13^	*U*^23^
N1	0.0289 (5)	0.0148 (4)	0.0242 (5)	−0.0005 (3)	−0.0016 (4)	−0.0007 (3)
O1	0.0287 (4)	0.0196 (4)	0.0387 (5)	0.0034 (3)	−0.0068 (4)	0.0017 (3)
C1	0.0231 (5)	0.0188 (5)	0.0234 (5)	0.0019 (4)	0.0001 (4)	0.0021 (4)
C2	0.0261 (6)	0.0176 (5)	0.0235 (5)	−0.0020 (4)	−0.0028 (4)	0.0005 (4)
C3	0.0220 (5)	0.0199 (5)	0.0265 (6)	−0.0006 (4)	−0.0044 (4)	0.0013 (4)
C4	0.0203 (5)	0.0189 (5)	0.0252 (6)	−0.0002 (4)	0.0010 (4)	−0.0002 (4)
C5	0.0211 (5)	0.0205 (5)	0.0242 (5)	−0.0030 (4)	0.0009 (4)	−0.0028 (4)
C6	0.0195 (5)	0.0244 (5)	0.0195 (5)	−0.0009 (4)	0.0000 (4)	0.0006 (4)
C7	0.0225 (5)	0.0167 (5)	0.0352 (6)	0.0008 (4)	−0.0001 (5)	−0.0008 (4)
C8	0.0311 (7)	0.0340 (7)	0.0540 (9)	0.0073 (5)	0.0090 (6)	0.0041 (6)
C9	0.0556 (9)	0.0304 (6)	0.0429 (8)	0.0106 (6)	0.0013 (7)	0.0108 (6)
C10	0.0369 (8)	0.0217 (6)	0.0866 (13)	0.0031 (5)	−0.0134 (8)	−0.0140 (7)
C11	0.0202 (5)	0.0308 (6)	0.0210 (5)	0.0006 (4)	−0.0017 (4)	0.0005 (4)
C12	0.0252 (6)	0.0406 (7)	0.0345 (7)	−0.0015 (5)	−0.0065 (5)	−0.0092 (6)
C13	0.0314 (7)	0.0525 (8)	0.0257 (6)	−0.0013 (6)	−0.0032 (5)	0.0105 (6)
C14	0.0218 (6)	0.0394 (7)	0.0285 (6)	0.0023 (5)	−0.0008 (5)	−0.0026 (5)
C15	0.0343 (6)	0.0166 (5)	0.0274 (6)	−0.0020 (4)	−0.0081 (5)	0.0016 (4)
C16	0.0291 (8)	0.0159 (7)	0.0233 (8)	0.000	−0.0030 (6)	0.000
C17	0.0370 (7)	0.0227 (6)	0.0333 (7)	0.0019 (5)	0.0059 (5)	−0.0046 (5)
C18	0.0523 (12)	0.0298 (9)	0.0286 (9)	0.000	0.0117 (9)	0.000

### Geometric parameters (Å, º)

**Table:**

N1—C16	1.4575 (13)	C10—H10A	0.9600
N1—C17	1.4719 (16)	C10—H10B	0.9600
N1—C15	1.4777 (14)	C10—H10C	0.9600
O1—C1	1.3770 (13)	C11—C14	1.5390 (17)
O1—H1	0.8200	C11—C12	1.5379 (17)
O2—H2	0.8500	C11—C13	1.5414 (17)
C1—C2	1.4062 (16)	C12—H12A	0.9600
C1—C6	1.4078 (15)	C12—H12B	0.9600
C2—C3	1.3909 (15)	C12—H12C	0.9600
C2—C15	1.5162 (15)	C13—H13A	0.9600
C3—C4	1.3962 (15)	C13—H13B	0.9600
C3—H3A	0.9300	C13—H13C	0.9600
C4—C5	1.3975 (16)	C14—H14A	0.9600
C4—C7	1.5359 (15)	C14—H14B	0.9600
C5—C6	1.4023 (16)	C14—H14C	0.9600
C5—H5A	0.9300	C15—H15A	0.9700
C6—C11	1.5414 (15)	C15—H15B	0.9700
C7—C10	1.5283 (17)	C16—N1^i^	1.4575 (13)
C7—C9	1.535 (2)	C16—H16A	0.9700
C7—C8	1.5335 (18)	C16—H16B	0.9700
C8—H8A	0.9600	C17—C18	1.5231 (16)
C8—H8B	0.9600	C17—H17A	0.9700
C8—H8C	0.9600	C17—H17B	0.9700
C9—H9A	0.9600	C18—C17^i^	1.5231 (16)
C9—H9B	0.9600	C18—H18A	0.9700
C9—H9C	0.9600	C18—H18B	0.9700
			
C16—N1—C17	110.29 (10)	C14—C11—C13	109.83 (10)
C16—N1—C15	110.62 (9)	C12—C11—C13	107.17 (10)
C17—N1—C15	112.13 (9)	C14—C11—C6	109.79 (9)
C1—O1—H1	109.5	C12—C11—C6	111.83 (10)
O1—C1—C2	119.33 (10)	C13—C11—C6	110.44 (10)
O1—C1—C6	119.81 (10)	C11—C12—H12A	109.5
C2—C1—C6	120.85 (10)	C11—C12—H12B	109.5
C3—C2—C1	119.74 (10)	H12A—C12—H12B	109.5
C3—C2—C15	119.80 (10)	C11—C12—H12C	109.5
C1—C2—C15	120.32 (10)	H12A—C12—H12C	109.5
C2—C3—C4	121.57 (10)	H12B—C12—H12C	109.5
C2—C3—H3A	119.2	C11—C13—H13A	109.5
C4—C3—H3A	119.2	C11—C13—H13B	109.5
C3—C4—C5	117.02 (10)	H13A—C13—H13B	109.5
C3—C4—C7	120.11 (10)	C11—C13—H13C	109.5
C5—C4—C7	122.87 (10)	H13A—C13—H13C	109.5
C4—C5—C6	124.06 (10)	H13B—C13—H13C	109.5
C4—C5—H5A	118.0	C11—C14—H14A	109.5
C6—C5—H5A	118.0	C11—C14—H14B	109.5
C5—C6—C1	116.73 (10)	H14A—C14—H14B	109.5
C5—C6—C11	121.28 (10)	C11—C14—H14C	109.5
C1—C6—C11	121.99 (10)	H14A—C14—H14C	109.5
C10—C7—C9	108.21 (12)	H14B—C14—H14C	109.5
C10—C7—C8	108.69 (11)	N1—C15—C2	112.34 (9)
C9—C7—C8	108.76 (11)	N1—C15—H15A	109.1
C10—C7—C4	112.50 (10)	C2—C15—H15A	109.1
C9—C7—C4	109.83 (10)	N1—C15—H15B	109.1
C8—C7—C4	108.78 (10)	C2—C15—H15B	109.1
C7—C8—H8A	109.5	H15A—C15—H15B	107.9
C7—C8—H8B	109.5	N1—C16—N1^i^	109.75 (13)
H8A—C8—H8B	109.5	N1—C16—H16A	109.7
C7—C8—H8C	109.5	N1^i^—C16—H16A	109.7
H8A—C8—H8C	109.5	N1—C16—H16B	109.7
H8B—C8—H8C	109.5	N1^i^—C16—H16B	109.7
C7—C9—H9A	109.5	H16A—C16—H16B	108.2
C7—C9—H9B	109.5	N1—C17—C18	109.51 (11)
H9A—C9—H9B	109.5	N1—C17—H17A	109.8
C7—C9—H9C	109.5	C18—C17—H17A	109.8
H9A—C9—H9C	109.5	N1—C17—H17B	109.8
H9B—C9—H9C	109.5	C18—C17—H17B	109.8
C7—C10—H10A	109.5	H17A—C17—H17B	108.2
C7—C10—H10B	109.5	C17^i^—C18—C17	110.30 (15)
H10A—C10—H10B	109.5	C17^i^—C18—H18A	109.6
C7—C10—H10C	109.5	C17—C18—H18A	109.6
H10A—C10—H10C	109.5	C17^i^—C18—H18B	109.6
H10B—C10—H10C	109.5	C17—C18—H18B	109.6
C14—C11—C12	107.71 (10)	H18A—C18—H18B	108.1

Symmetry code: (i) *x*, −*y*+3/2, *z*.

### Hydrogen-bond geometry (Å, º)

**Table:**

*D*—H···*A*	*D*—H	H···*A*	*D*···*A*	*D*—H···*A*
O1—H1···N1	0.82	2.00	2.6880 (13)	142
O2—H2···O1^i^	0.85	2.22	3.036 (5)	161

Symmetry code: (i) *x*, −*y*+3/2, *z*.
